# A model framework to communicate the risks associated with aflatoxins

**DOI:** 10.1038/s41538-023-00217-y

**Published:** 2023-08-11

**Authors:** Kiran Bhardwaj, Julie P. Meneely, Simon A. Haughey, Moira Dean, Patrick Wall, Awanwee Petchkongkaew, Bob Baker, Guangtao Zhang, Christopher T. Elliott

**Affiliations:** 1 Institute for Global Food Security, School of Biological Sciences, Queen’s University Belfast, Belfast, BT9 5DL Northern Ireland United Kingdom; 2 University College Dublin, School of Public Health Physiotherapy and Sports Science, Woodview House Belfield Dublin 4, Dublin, Ireland; 3 School of Food Science and Technology, Faculty of Science and Technology, Thammasat University, Bangkok, Thailand; 4 Mars Global Food Safety Research Center, Bejing, China

**Keywords:** Agriculture, Risk factors

## Abstract

Risk communication is defined as the interactive exchange of information and opinions concerning risk, risk-related factors and risk perceptions amongst all the stakeholders of food safety throughout the risk analysis process. The interactive exchange of information occurs at three different levels i.e. informed level, dialogue level and engagement level. For an effective food safety risk communication (FSRC), it is important that the information should adhere to the core principles of risk communication which are transparency, openness, responsiveness and timeliness. Communication of a food safety risk within all the components of risk communication strategy constitutes a complex network of information flow that can be better understood with the help of a framework. Therefore, a model framework to communicate the risks associated with aflatoxins (AFs) dietary intake has been developed with the aim of (a) creating general awareness amongst public and (b) involving industry stakeholders in the prevention and control of risk. The framework has been motivated by the learnings and best practices outlined in the identified technical guidance documents for risk communication. Risk assessors, risk managers, industry stakeholders and general public have been identified as the major stakeholders for the present framework. Amongst them, industry stakeholders and general public has been selected as the major target audience for risk managers. Moreover, population residing in low- and middle-income countries (LMIC) has been identified as the main target group to reach.

## Definition, goals and principles

Risk communication is defined as ‘the interactive exchange of information and opinions concerning risk, risk-related factors, and risk perceptions among stakeholders including risk assessors, risk managers, feed and food industries, general public and other interested parties (academia, media and NGOs, etc) throughout the risk analysis process’^1,2^. The interactive exchange of information occurs at three different levels i.e. informed level (public awareness), dialogue level (information exchange between two stakeholders such as risk assessors and risk managers) and engagement level (involvement of all the stakeholders in decision making)^1,2^. Each of these levels differ in terms of communication requirements such as technicality of information, channels used, timing and frequency of the information exchange. The ultimate goal of food safety risk communication (FSRC) is to safeguard consumer health by providing information regarding the rationale behind decisions made and actions taken to all the stakeholders of food safety. Thus, creating a better understanding and dialogue amongst them and enabling people to make informed judgements about food safety risks^1^.

For an effective FSRC strategy, it is critically important that the communicated message builds trust with the public as they may not believe or follow what they can’t rely upon. People tend to trust credible information sources which demonstrate a good understanding of the communicated risk, understand their concerns/perceptions and provide them with unbiased conclusions and recommendations. In addition to this, people also seek expertise of the sources in assessing, managing and communicating the risks which helps in building confidence in the source of information^1^. Lack of confidence and trust in the sources can result in negative public reaction as a result people may start following other sources such as some profit minded organisations or politically driven campaigns leading to poor risk management in terms of misinformation/miscommunication spread, especially through social media channels. Therefore, in order to create trustworthiness and to avoid miscommunication, risk information should be undertaken in an open, transparent, responsive and timely manner which are the core principles of risk communication. Best practices to adhere to the above principles are mentioned below.

## Transparency and openness

Transparency refers to providing evidence and the rationale behind the decision making while openness refers to the opportunity of engaging all stakeholders and interested parties in the decision-making process and together with transparency help builds trust amongst the audience^1–4^. In practice, trust with transparency and openness can be achieved by the following.

### Conveying clear, concise and understandable information to avoid miscommunication/misinterpretation of risk

Scientific information should be explained in a simple language so that non-technical audiences like the general public can understand its relevance and should be able to use it for the correct purposes^1–4^. For example, distribution of infographics, leaflets, factsheets and telecasting of short informational videos and messages via social or traditional media platforms can achieve high levels of general awareness as observed in the communication of risk posed by zoonotic diseases in European Union (EU)^3,5^. Additionally, awareness programme regarding risk posed by high salt intake in the UK’s adult population also received positive outcomes, in terms of reduced salt intake and an increased number of consumers cutting down on their salt intake, by the means of the above-mentioned tools and channels used as communication mediums in the Salt Campaign^3,6^.

### Providing access to all the key documentations and scientific outputs to key audiences

Openness and transparency are important for building trust and can be achieved by publishing documents (minutes of meetings, research reports, etc) on a website to create awareness and understanding amongst stakeholders^1–4^. For example, during the Irish dioxin crisis of 2009 and 2010 that arose from the contamination of pork products, the Food Standard Authority of Ireland’s (FSAI) used high level media coverage, information sharing through its website and an advice line to recall all implicated pork products from the shelve. They also gave some advice to households to dispose of or return affected products to retailers. This resulted in a positive outcome as all the implicated products were removed and uncontaminated products were put back in the markets within 6 days^3,7^.

The risk communication of preventive measures introduced by the Dutch government to kill infected goats to stop the spread of Q-Fever is another example of open and transparent risk communication. High-level media coverage was used to communicate the decision with the aim of showing government’s concern for animal welfare and goat farmers which resulted in a positive impact in terms of gaining a high level of understanding from farmers. It is also a good example of stakeholder engagement as opinions from involved stakeholders (Dutch agency, media, press and veterinarians) and co-operation amongst them was considered as paramount to achieve high levels of understanding from farmers over the communication of such an emotive operation, i.e., culling of infected goats^3,8^.

### Facilitating two-way communication and stakeholder engagement in decision making process

Dialogue with stakeholders and engagement can be achieved by inviting them to provide evidence, participate in decision making process and to provide feedback on message drafts and concerns raised after communicating the risks. This provides risk managers a better understanding of needs and concerns of the target audience which is essential for a good risk communication. Stakeholder engagement also facilitates the understanding of risk perception by the target audience which is also crucial for effective communication^1–4^. Risk assessment on animal cloning^3,9^ was a decision that was made following a wide range of media engagement and dialogue with stakeholders, i.e., public consultations.

Another example of using dialogue with stakeholders to identify the risk perceptions and concerns regarding health can be seen in the communication of Chaga’s disease in Brazil. Educational campaigns were run for street venders and food producers by developing messages tailored to risk perceptions related to health concerns of not implementing Good Manufacturing Practices (GMP)^1,10^. Communications in Africa regarding AF contamination used public opinion surveys as a medium of identifying risk perceptions and information needs of the target audience^1,11^.

## Responsiveness and timeliness

Responsiveness refers to the extent to which risk managers address concerns and expectations of target audience while considering gaps or uncertainties about the food safety risk. Responsiveness can also build trust amongst audience by the following.

### Communicating accurate and appropriate information in a timely manner

Timely communication of the food safety issue, even when all the facts are not known, is essential to build trust and confidence in the long term. Therefore, it is recommended that communication should start from early stages (explaining the conclusions of risk assessments, decision making process with the knowledge of possible measures) and continue till the end, explaining the decision and the rationale behind it. If timeliness is compromised, then it can lead to erosion of trust amongst the audience^1–4^. For example, a decision taken by the Public Health Agency of Canada (PHAC) not to communicate the outbreak of listeriosis until the source of illness was confirmed, which took 10 days after the outbreak, attracted widespread criticism from public and reduced government’s credibility and perceived competence in risk management. The government was accused by media of putting manufacture’s interest above that of public health. Consequently, public lost trust in the government and all the further communications were ineffective. Therefore, communications regarding significant health risks should be made in a timely manner i.e. as soon as the risk assessment conclusions were made, even when there are uncertainties in the assessments^1,12^.

### Acknowledging and explaining the uncertainties of risk assessments

For risk communications to be timely and transparent, it is imperative to acknowledge and address the uncertainties in risk assessment results. Food safety issues with significant health impacts, like food borne illness, AF-related health effects, require early communications otherwise this could lead to increased public health risks and erosion of public’s trust in government^1–4^, as observed in the case of listeriosis outbreak in Canada. Despite the uncertainty regarding the source of illness, the Canadian government could have spread general awareness about what preventive measures could be taken by public to protect themselves from the infection. This could have reduced some of the cases that occurred^1,12^. This can be taken as an important learning for the communication of AFs as some degree of uncertainty could be observed regarding the risks posed by dietary intake of AFs through contaminated commodities. Therefore, public should be informed that there is a potential risk associated however less data is available to estimate the extent or severity of risk.

### Understanding target audience

For an effective risk communication, it is important for risk managers to understand needs, concerns and risk perceptions of the target audience. Communications that are not tailored to the needs of these and do not acknowledge their concerns and perceptions of risk are not trusted by audiences and can result in ineffective risk management^1–4^. For example, in 2012 in the USA, the American consumer advocacy magazine Consumer Reports and the Food and Drug Administration (FDA) both published findings of significant levels of inorganic arsenic found in the tested rice and rice products. The results of the sources were largely consistent with each other, but consumer reports attracted large media coverage and significant interest from public than the FDA’s report, reason being the significant difference in their recommendations to the public. Consumer reports advised public about the preventive measures to limit their exposure to arsenic, differentiating among infant, adults and the elderly. On the other hand, the FDA advised public to continue eating rice and rice products and stated that it would be early to recommend dietary changes as a rigorous risk assessment was needed and did not recommend any actions by which public could limit their exposures. Additionally, the agency failed to address the concerns of people who were medically or culturally dependant on rice-based diet and to acknowledge the potential risk to infants and pregnant women. The FDA’s approach of not addressing public concerns and perceptions led consumers to turn to the specific guidance provided by consumer reports^1,13^. The FDA could have advised public to limit their exposures while explaining the uncertainties about scientific data to recommend specific dietary changes. On the other hand, communications of AFs in Africa and risk assessment of animal cloning in EU have shown good examples as the awareness and educational campaigns were designed after understanding the risk perceptions and needs of the target audience by conducting public surveys or consultations^1,10,11^.

Therefore, it can be learnt that risk communication regarding food safety hazards with high public concern such as AFs, risk management should consider the public’s perception of risk as people generally tend to perceive lower degrees of risk from naturally occurring hazards such as AFs which is mainly due to low level of understanding, awareness and knowledge regarding the severity and likelihood of the risk, especially in LMICs. It could also be because AFs are unobservable in nature and mostly have chronic health consequences, especially liver cancer. In order to increase consumers’ beliefs and risk perception regarding threats posed by AFs, risk communication should involve region specific data on AF incidence and strategies to limit AF exposure by recommending dietary changes while addressing the needs and concerns of people depending on AF contaminated food^1^.

## Framework

**Fig. 1:**
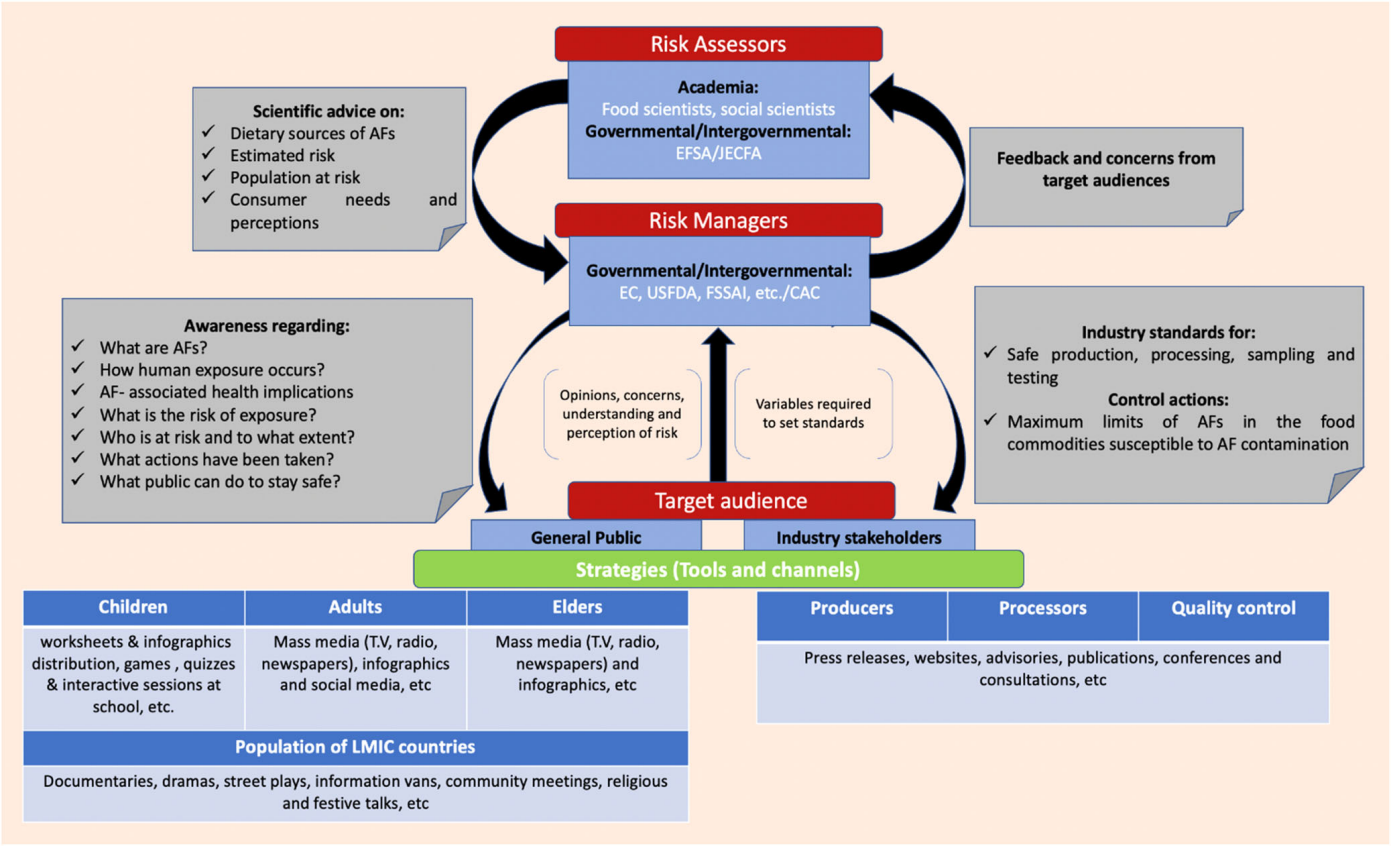
Model framework. Proposed risk communication framework for communicating the risk associated with AFs amongst relevant stakeholders via various channels and tools.

Communication of a food safety risk within all the components of risk communication strategy constitutes a complex network of information flow which can be better understood with the help of a framework. In 2021, Mars, Incorporated convened the Food Safety Coalition of like-minded individuals from industry, academia, and international organisations to drive food safety insights and best practices at pace, starting with aflatoxins due to serious health threat they pose. Work is being progressed in four areas: sampling and testing, risk assessment and communication, prediction, and risk communication. This publication forms part of the work focused on risk assessment and communication which was further divided into Part A: Risk assessment and Part B: Risk Communication. The present study covers Part B: Risk communication with an objective to develop a model framework (Fig. 1) for risk managers to communicate the risks associated with AFs dietary intake with the aim of (a) creating general awareness amongst public/consumers and (b) involving industry stakeholders (producers, processors, and quality assurance) in the prevention and control of risk. The framework has been developed in accordance with core principles of risk communication (*see Definition, goals and principles*) and has been motivated by the learnings and best practices described in the identified technical guidance documents, providing a basis for the present risk communication framework, which included:

1. ‘The application of risk communication to food standards and safety matters’, Food and Agriculture Organisation/World Health Organisation (FAO/WHO), 1998^14^
2. ‘Risk communication applied to food safety handbook’, FAO/WHO, 2016^1^
3. ‘When the food is cooking up a storm’, European Food Safety Authority (EFSA), 2018^3^
4. ‘Technical assistance in the field of risk communication’, EFSA (2021)^2^
5. ‘APEC Food Safety Risk Communication Framework and Associated Guidelines’, Hong Jin (Food Standards Australia New Zealand) and Amy Philpott (Watson Green LLC) on behalf of Food Standards Australia New Zealand (FSANZ), 2022^4^

All the components of the framework facilitating a continuous flow of information via various tools and channels within the food safety system are described below.

## Roles and responsibilities

### Risk assessors

Scientific experts from different academic and research institutions should act as the risk assessors in the FSRC strategy and serve as the independent source of information for risk managers, providing scientific opinions regarding food safety concerns and public health^14^. In the case of AFs (Fig. 1), food scientists from different universities, research institutions and governmental panels such as EFSA are responsible to act as risk assessors providing scientific expertise on major dietary sources and venerable population groups identified by conducting risk assessments for dietary intake of AFs through different food commodities. In addition to this, intergovernmental bodies like Joint FAO/WHO Expert Committee on Food Additives (JECFA) are also responsible for evaluating the risks of consuming certain food additives and contaminants, including aflatoxins. JECFA is an independent committee of scientific experts, which conducts risk assessments and provides scientific advisory for the risk managers. JECFA’s risk assessment reports are reviewed by national/regional governmental legislative bodies of many countries and some intergovernmental bodies like the Codex Alimentarius Commission (CAC), when establishing control measures^15^. Moreover, social scientists who study stakeholder perceptions, needs and concerns should also be involved to provide scientific advice on effective risk communication approaches and strategies^14^. Therefore, risk managers will be the key audience for risk assessors with technical level of scientific literacy and understanding^2^.

### Risk managers

Governments as policy makers are fundamentally responsible to act as risk managers for the communication of a clear, relevant, and accurate food safety risk information to all the stakeholders and interested parties in a timely manner, starting from early stages of assessment to the end of decision making^4^. Risk managers are also responsible to plan an effective risk communication strategy based on the core principles of risk communication (*see Definition, Goals and Principles*) that may differ according to the food safety issues, level of understanding and knowledge of food safety, risk communication objectives, and target audience^14^. For AFs (Fig. 1), legislative bodies such as the European Commission (EC), the US-Food and Drug Administration (USFDA), the Food Safety and Standards Authority of India (FSSAI), etc are the risk managers with the prime role of communicating decisions regarding sampling and testing procedures, safe handling and storage procedures and Maximum Limits (MLs) for the occurrence of AFs in susceptible food commodities to industries and spreading general awareness amongst general public. Additionally, CAC as an intergovernmental body is also responsible for the development of science-based industry standards and establishing MLs. Standards and MLs proposed by CAC are voluntary in nature and serves as a reference guidance documents for many national/regional governmental legislative bodies in establishing control measures^15^. Therefore, industry stakeholders and consumers/general public have been identified as major target audience for the present framework.

### Industry stakeholders

Industry stakeholders are primarily responsible for maintaining the quality and safety of the food products to safeguard the health of consumers^4^. Many countries have laid down laws and regulations that are required to be follow by food business operators to market safe and wholesome food products. For example, the General Food Law in EU (Regulation (EC) No 178/2002) lays down general principles, requirements and procedures of safe food and feed production and distribution by the establishment of European Food Safety Authority (EFSA)^16^. In the USA, the FDA has laid down regulations for the preventive controls and regulations for safe food production, packaging, storage, transportation and import under the Food Safety Modernisation Act (FSMA)^17^. Additionally, the Food Safety and Standards Act, 2006 enables FSSAI to lay down regulations for safe manufacturing, storage, distribution, sales and import, ensuring the availability of safe and wholesome food to consumers^18^. However, roles and responsibilities may differ according to different food business sectors., Producers and processors must ensure safe food production, handling and storage by the implementation of Good Agricultural Practices (GAP’s), Good Hygienic Practices (GHP’s) and Good Manufacturing Practices (GMP’s) throughout the food supply chain to prevent AF contamination during pre-harvest and post-harvest stage. Whereas quality assurance personal would ensure that the food being produced and marketed is safe for human consumption by testing the occurrence of AFs in susceptible food commodities.

### General public/consumers

Consumers play a major role in measuring the effectiveness of the communication strategy by the expression of their opinions, perceptions, concerns and understanding of the communicated food safety issue^4,14^.

### Levels of information exchange

The interactive exchange of information amongst stakeholders occurs at three following levels:

### Informed level

An Informed level of communication is defined as the dissemination of information amongst the audience to create understanding and awareness of the food safety issue, what is being done to mitigate it and what further actions are required from the audience to improve public health^1,2^. In the case of AFs (Fig. 1), risk managers should communicate all the findings to industry stakeholders and general public, thus creating an informed level of information exchange. However, the information may differ for both of target audiences and is explained in a later section.

### Engagement level

This type of communication facilitates the involvement of all the stakeholders in decision making regarding food safety issue^1,2^. In the present case (Fig. 1), risk managers being the policy makers are solely responsible to ensure the continuous involvement risk assessors, industry stakeholders and general public in making policies regarding AFs. For this a dialogue between risk managers and all the other stakeholders is required.

### Dialogue level

A dialogue level communication allows the exchange of information and ideas between two different stakeholders^1,2^. Dialogue provides risk managers with the important and relevant information required for risk assessments or risk management and increase the chances of making decisions which are fit for the purpose. In the present scenario (Fig. 1), a dialogue between risk assessors and risk managers should occur by co-ordinating the outcomes of research including dietary sources of AFs, what is the estimated risk and who is exposed to the risk (from risk assessors to risk managers) and the public concerns/perceptions raised after the communicating the risk (from risk managers to risk assessors, especially to those involved in social sciences). Additionally, a dialogue between risk managers and Industry stakeholders is also important for the making of industry standards for production, processing, and testing as industry personals are directly connected to food processing and handling system and can provide better understanding of the variables and capabilities required to set those standards. Moreover, dialogue level of communication should also take place between risk managers and the general public with regards to their opinions, perceptions and understanding of the communicated risk to which government acknowledge, respond and communicate them to risk assessors.

### Target audience

The FSRC strategy involves a complex system that works for the sole objective of communicating risk to people who have many different concerns^1^. Therefore, it is important that the communication message should be tailored to the needs of the key audiences which further implies the need to segment audience based on factors such as the type of risk, socio-cultural and demographic factors^1,2^. Audience segmentation provides a better understanding of the needs of target audiences prior to the development of communication message. Taking insights from the EFSA’s mapping model that categorizes key audience into three segments i.e. “entry”, “informed” and “technical” level^2^, Industry stakeholders (technical level) and the general public (entry level) were identified as the two key target audiences for the present framework (Fig. 1). Industry stakeholders have been segmented into producers, processors, and quality assurance managers while general public has been segmented based on their age, according to which children, adults and the elderly have been identified as the major segments. Amongst them, population residing in low- and middle-income countries (LMIC) has been identified as the main target group to reach as most of the risk associated with AFs lies in LMIC with no or less awareness amongst general public regarding AFs.

## Communication message design process

### Development

Once the audience is segmented then it is time to develop and present appropriate and effective messages that may vary across the identified target audiences based on the communication objective and their needs. For example, in case of AFs (Fig. 1), for technical audiences like risk managers and industry stakeholders, specifics regarding timelines of risk assessments, results of risk estimations based on Margin of Exposures (MOEs) and liver cancer potency, industry standards and maximum limits on the occurrence etc, may be of more interest. However, general public would be interested to know the nature of risk, who/what is going to be affected and to what extent (severity), type of exposure, i.e., acute or chronic exposure and what control and preventive measures to be taken by them to stay safe. Therefore, well-targeted messages presented in a non-technical language would be more effective for general public^3^.

Message content would also differ across different stages of the risk analysis depending upon the objective and target audience^2^. A summary of the content type required to be developed for each stage in accordance with the communication objective and audience type has been provided by EFSA^2,19^, according to which the content would differ for framing, assessment, evaluation and management stage as the objective and key audience would be different for these stages. For example, the objective of assessment stage is to exchange information about the characterised risk mainly with technical stakeholders and general public as the key audiences. Therefore, the message content should describe sources of information, milestones, and timeline of the process^2,19^.

In addition to this, the appropriateness of the message content may also vary according to the subject area of the food safety risk^2^. For example, risk communication for microbial hazards would be based on the risk perceptions and lifestyle of the audiences^2,20^. Whereas ethics and values would play a greater role for the area of animal health and welfare would. Based on these factors (target audience, risk analysis stages and subject area), the communication objectives and requirements for the present framework have been given in the Table 1.

**Table 1:** Communication objectives and requirements for AFs risk communication framework, adapted from EFSA^2^ and Renn^19^.

Risk analysis stage	Source	Communication objective	Key audience	Level of information	Content requirements
Assessment	Risk assessors	To share scientific opinions and outcomes of research To advice on effective communication strategies by studying feedbacks, concerns and risk perceptions from stakeholders and public.	Risk managers	Dialogue	Technical
	Risk managers	Transparent exchange of facts and arguments concerning risk assessment and/or characterisation process	Industry stakeholders	Informed	Technical
			General Public	Informed	Non-technical, Visual aids, Aids for people with disabilities and low literacy levels
Evaluation	Industry stakeholders	To provide feedback on the undertaken processes	Risk managers	Engagement	Technical or non-technical
	General public	To express concerns and perceptions regarding processes			
	Risk managers	To present feedbacks and concerns from industry stakeholders and general public.	Risk assessors	Dialogue	Technical or non-technical
Management	Risk managers	To share guidance and compliance documents for effective, efficient, fair, ethical and feasible production, processing and testing of food commodities susceptible to AF contamination	Industry stakeholders	Informed	Technical
		To raise awareness amongst consumers by providing information regarding risk, exposure levels, severity and actions to be taken to keep them safe.	General public	Informed	Non-technical, Visual aids, Aids for people with disabilities and low literacy levels

### Presentation and framing (mode of delivery)

Presentation and framing are two key aspects that need to be considered as the part of content design process. The way key messages are presented has a great impact on risk perception amongst target audiences. Therefore, to avoid misinterpretation and misperception of scientific findings it is important that facts in the message should be carefully framed and presented in a way that is of audience’s interest and should be conveyed in a clear and understandable manner^1^. For example, the young generation that have low food safety knowledge and usually do not pay much attention to food safety, a brief, eye-catching and engaging message is required to be developed to create basic understanding and awareness of the issue^2,21^. In addition to this, learnings from the communication regarding AFs in Africa suggests that location specific food safety hazard data and strategies to manage the hazard can be incorporated in the message to increase public interest and attention in the ongoing risk communication^1,11^.

The messages should also stick to the principle of ‘STARC’ i.e. Simple, Timely communicated, Accurate, Repeated and Consistent^1^. Additional considerations for message presentation for general public include:

- The usage of Visual aids such as graphics, diagrams and illustrations, especially for immigrants, tribes unfamiliar with language and people with low literacy rates^1^.
- The needs of individuals sight, hearing, speech and other disabilities. The message should reach all the target audience and must meet their needs^1^.

### Medium of delivery (Tools and channels)

The last step in the content design process is to choose the best suitable medium of delivery (tools and channels) for the delivery of developed risk communication messages. The selection of appropriate and effective tools and channels depends on following factors^1^:

- Objective of risk communication
- Content or nature of the message
- Accessibility
- Use by target audience

Tools can be any (i) product and service (press releases, websites, videos, publications) and/or (ii) Methods and approaches (interviews, polls, questionnaires) used to deliver/support the information exchange and to understand the communication preferences of the audience, respectively. Whereas, channels are defined as the means for reaching out to those products and services. The tools have been categorised as:

- **Information based**: used to inform a large number of audiences, e.g. written material, newspapers, press releases, websites, events, etc.^2,22^.
- **Dialogue based**: used to establish two-way communication by involving audiences in Q/A sessions and discussions. Examples include chat rooms, opinion polls, open days for visitors, leaflets with return coupon and panel discussions^2,22^.
- **Participation based:** used to integrate the concerns of audience into the decision-making process. For example, orientation tools (focus groups, citizen assemblies and hearings), self-governing tools (working groups and round tables) and decision-making tools (conferences)^2,22^.

On the other hand, channels can be divided into two broad categories including traditional media and social media channels. Traditional media channels usually facilitate one-way communication of the risk and include T.V., radio, newspapers. While social media channels provide two-way form of communication^2,23^. Amongst all, T.V. and radio (70%) remained most common and most effective communication channels among US consumers^2,24^. A special Eurobarometer^2,25^ on food safety reported T.V. (69%) as the most common source of information about food related risks in the EU, followed by internet (excluding social media) (46%) and newspapers and magazine (38%). Also, preference for the sources of information may vary according to age. For example, youngsters (15–24 years old) in the EU were more likely to prefer social media (45% vs. 10%) for food-related information than older people (aged 55 or over). On the other hand, older people preferred television (78% vs. 55%), newspapers and magazines (46% vs. 22%) and radio (30% vs. 13%) than youngsters^2,25^. Therefore, social media platforms will be best suitable to reach young audiences, while traditional media channels would be more appropriate to reach older people.

In addition to the traditional and social media channels, people can also act the sources of information, especially when communicating to the general public. For the source of information to be credible, informed and trusted, it is required that the information should come from a trusted independent body^2^. Scientists (82%) and consumer organisations (79%) are considered as the most trusted sources amongst consumers in the EU^2,25^. For the effectiveness of the risk communication strategy, it is recommended to use multiple channels including traditional media, social media and consumer organisation as the chances to reach and engage the audience are increased due to the combined strengths of each medium^2^.

Tools and channels suitable to communicate risk associated with AFs, identified based on their proven effectiveness in the previous risk communications, have been mentioned in Table 2. Best practice tools and channels to reach and engage industry stakeholders in the AF risk communications have been identified as press releases, websites, publications, advice lines, public consultations, conferences, meetings and Q/A sessions. In general, infographics, leaflets, and factsheet distribution, mass media (T.V., radio, Newspapers) coverage for one-way communication while social media (twitter, facebook, LinkedIn, etc) coverage for two-way communication, have been identified as best suitable tools and channels for AF risk communication targeting all audiences in general public. Awareness amongst children can be spread by distributing infographics, fact sheets, leaflets and conducting interactive guest lectures at schools. Additionally, students can be asked to participate in games and quizzes related to AFs. For communications adults, mass media and social media coverage of the food issue has been proven effective to spread awareness regarding AFs.

**Table 2:** Best practice tools and channels for risk communication regarding AFs.

Target Audience	Best Practices (Tools and channels)		Case studies
	One way communication	Two-way communication	
**Industry stakeholders**	Press release, websites, Publications, advice lines, etc	Public consultations, conferences, meetings and Q/As, etc	• Acrylamide in food^26^ • Risk assessment on animal cloning^9^ • Irish dioxin crisis^7^
**General public**			
**All audience in general**	Fact sheets distribution, Leaflets, T.V, radio, newspaper articles, etc	Infographics and videos on social media (twitter, LinkedIn, Facebook, youtube,) and websites with commenting options.	• Acrylamide in food^26^ • Communication on food-borne zoonotic diseases^5^ • Q-fever in the Netherlands: openness and Transparency^8^
**Children and adolescents**	Leaflets factsheets distribution in schools, and worksheets, etc	Games, quizzes, social media, and interactive guest lectures, etc	• Young et al. ^27^
**Adults and elders**	T.V. and radio advertisements, posters, newspapers, and leaflets, etc	Social media	• Salt campaign^6^
**Low/middle income countries**	Radio Jingles, newspapers, Documentaries, communication via information services (Ex vans), dramas, and street plays, etc	Talks at religious gatherings or festivals, Quiz competitions, and community meetings, door-to-door awareness, etc	• India’s National Aids Control Programme^28^ • Accessing Senegal’s anti-Aids Successes^29^ • Aflatoxin contamination of staples in Africa^11^

Amongst the general public, population of low-middle income countries has been identified as the major target group to reach for the present framework because of the high level of occurring AF contamination and low level of knowledge and awareness amongst population. Due to the diversity in the population of LMICs, there is a possibility to neglect and/or exclude some people that are hard to reach such as population with low literacy levels and/or population that do not have access to the social media platforms^1^. Therefore, in addition to the above-mentioned conventional media, some unique communication tools and channels such as role playing at market centres, community-based communications (community meetings, workshops and talks in markets, schools and religious places), radio jingles and television documentaries have also been identified as suitable mediums that are particularly relevant to the population of LMICs (Table 2).

## Discussion

To conclude, this review has illustrated some very good but also some less optimimal ways that risks have been communicated to stakeholders. As a general summary we have selected some important do’s and don’ts to help guide those embarking on risk communication potentially for the first time (Table 3). It has been demonstrated that risk communication strategy constitutes a complex network of information flow within a framework and that the this flow should be based on the core principles of risk communication (transparency, openness, responsive and timeliness). The current review presented a framework for AF related risk communication (Fig. 1) including risk assessors, risk managers, industry stakeholders and general public as the major stakeholders. Different stages of information exchange within the developed framework occurs at three levels i.e. informed level (communication of findings by risk managers to industry stakeholders and general public); engagement level (involvement of all the stakeholders by risk managers in policy making) and dialogue level (exchange of information amongst risk assessors, risk managers, industry stakeholders and general public regarding outcomes of assessment, industry standards and public’s perception of risk). For an effective risk communication, it is important the communication message should build trust otherwise the audience may start following alternative sources, increasing chances of miscommunication. Trust can be built by clear, concise, responsive and timely communication of the risk that should be tailored to the needs and concerns of the target audience. Industry stakeholders (technical) and general public (non-technical), especially public in the LMICs have been identified as major target audience. The target audience an important role in the communication design process. Messages are developed and framed as per their level of understanding and therefore includes technical and detailed information for the industry stakeholders whereas simple, concise and clear messages are developed and presented to the general public. Apart from this, tools and channels for message delivery also vary according to the target audience. Industry stakeholders can be reached by press releases, websites, conferences and meetings, etc. However, distinctive communication channels have been identified for the general public including fact sheets, leaflets, infographics, mass media and social media channels, etc. Some special channels including documentaries, street plays, community meetings and religious talks, etc have been identified for the populations of LMIC.

**Table 3:** Do’s and Don’ts of risk communication.

Stage	Do’s	Don’ts
**Audience segmentation**	Understand needs, concerns and risk perceptions of the target audience.	Do not ignore the needs, concerns and risk perceptions of the target audience as it can lead to lack of interest in the target audience which consequently result in an ineffective risk management.
	Consider the needs of audience with low literacy levels and disabilities.	Do not ignore the audience with special needs while designing and communicating risk communication information for the better risk management.
**Designing risk communication message**	Frame messages tailoring to the needs of the target audience.	Do not frame generalised messages as it can result ineffective risk communication.
	Frame clear, concise and simple messages.	Avoid framing complex messages to prevent miscommunication or misinterpretation.
**Communicating risk**	Communicate as early as possible and continue till the end.	Avoid late communications and discontinuity as it can create confusion and reduce audience’s trust in the management.
	Provide access to the key documents and rationales behind decision making.	Do not leave audience with assumptions regarding decision making processes as it can lead to misinterpretation and lack of trust amongst them.
	Engage audience by facilitating two-way communication.	Avoid one way communication and try to engage audience for the better understanding of their needs and concerns.
	Acknowledge and address uncertainties in the risk communication.	Do not ignore uncertainties in the risk communication as it can again result in erosion of public’s trust and confidence.

## Data Availability

Data sharing is not applicable to this article as no data was generated or analysed during the current study.
